# Continuous positive airway pressure and ventilation are more effective with a nasal mask than a full face mask in unconscious subjects: a randomized controlled trial

**DOI:** 10.1186/cc13169

**Published:** 2013-12-23

**Authors:** Jun Oto, Qian Li, William R Kimball, Jingping Wang, Abdolnabi S Sabouri, Priscilla G Harrell, Robert M Kacmarek, Yandong Jiang

**Affiliations:** 1 Department of Anesthesia, Critical Care, and Pain Medicine, Massachusetts General Hospital, 55 Fruit Street, Boston, MA 02114, USA; 2 Department of Anesthesia, West China Hospital of Sichuan University, 37 Guoxuexiang, Chengdu, Sichuan 610041, China; 3 Respiratory Care Services, Massachusetts General Hospital, 55 Fruit Street, Boston, MA 02114, USA

## Abstract

**Introduction:**

Upper airway obstruction (UAO) is a major problem in unconscious subjects, making full face mask ventilation difficult. The mechanism of UAO in unconscious subjects shares many similarities with that of obstructive sleep apnea (OSA), especially the hypotonic upper airway seen during rapid eye movement sleep. Continuous positive airway pressure (CPAP) via nasal mask is more effective at maintaining airway patency than a full face mask in patients with OSA. We hypothesized that CPAP via nasal mask and ventilation (nCPAP) would be more effective than full face mask CPAP and ventilation (FmCPAP) for unconscious subjects, and we tested our hypothesis during induction of general anesthesia for elective surgery.

**Methods:**

In total, 73 adult subjects requiring general anesthesia were randomly assigned to one of four groups: nCPAP P0, nCPAP P5, FmCPAP P0, and FmCPAP P5, where P0 and P5 represent positive end-expiratory pressure (PEEP) 0 and 5 cm H_2_O applied prior to induction. After apnea, ventilation was initiated with pressure control ventilation at a peak inspiratory pressure over PEEP (PIP/PEEP) of 20/0, then 20/5, and finally 20/10 cm H_2_O, each applied for 1 min. At each pressure setting, expired tidal volume (Vte) was calculated by using a plethysmograph device.

**Results:**

The rate of effective tidal volume (Vte > estimated anatomical dead space) was higher (87.9% vs. 21.9%; *P*<0.01) and the median Vte was larger (6.9 vs. 0 mL/kg; *P*<0.01) with nCPAP than with FmCPAP. Application of CPAP prior to induction of general anesthesia did not affect Vte in either approach (nCPAP pre- vs. post-; 7.9 vs. 5.8 mL/kg, *P* = 0.07) (FmCPAP pre- vs. post-; 0 vs. 0 mL/kg, *P* = 0.11).

**Conclusions:**

nCPAP produced more effective tidal volume than FmCPAP in unconscious subjects.

**Trial registration:**

ClinicalTrials.gov identifier: NCT01524614.

## Introduction

Mask ventilation as an initial ventilation support is widely used for unconscious subjects either in an emergency or during induction of general anesthesia [1]. About 250,000 cases of cardiac arrest annually occur outside of hospitals, and 370,000 to 750,000 cases occur in hospitals [2,3]. The total number is close to one million emergency mask ventilations performed per year. In addition, about 21 million cases are performed under general anesthesia (GA) and the majority require mask ventilation during induction of GA [4].

Mask ventilation is nearly exclusively provided by using a full face mask [1]. Unfortunately, full face mask ventilation is difficult to master, and the skill is hard to retain without frequent re-enforcement [5,6]. The rescuer has to ensure adequate mask seal, head placement, and lower jaw advance with one hand and perform bag ventilation with the other hand [7]. Given that the need for emergency mask ventilation can occur anytime and anywhere, in the hospital, in public places, and at home, the ability of medical personnel and the lay public to perform adequate emergency ventilation is far from satisfactory.

The mechanism of upper airway obstruction (UAO) in unconscious victims is not fully understood. However, it shares many similarities with that of obstructive sleep apnea (OSA) [8]. Continuous positive airway pressure (CPAP) is the standard treatment for maintaining upper airway patency in patients with OSA [9]. If the patient is able to tolerate CPAP and use it correctly, its effective rate is nearly 100% [10,11]. The majority of patients with OSA fail the treatment not because of its low efficacy for maintaining upper airway patency but because of intolerance [12]. In addition, CPAP via nasal mask is more effective at maintaining airway patency than a full face mask in patients with OSA [9,13]. Treatment with CPAP for patients with OSA usually starts before patients fall asleep and the occurrence of UAO; however, it is unclear whether the efficacy of CPAP in treating OSA is reproducible for unconscious victims after UAO develops.

Recently, we demonstrated that applying ventilation through both the mouth and nose was less effective than through the nose alone and that ventilation occurs primarily through the nasal route even when both routes are used [14]. We also reported that direct mouth-to-nose breathing is more effective than mouth-to-mouth breathing in unconscious adult subjects with apnea [15]. Because the high success rate of reducing UAO in patients with OSA results not only from the nasal mask but also from employing CPAP, we hypothesized that CPAP via nasal mask and ventilation (nCPAP) should be more effective in reducing UAO for unconscious victims than CPAP via full face mask and ventilation (FmCPAP). Because treatment with CPAP for patients with OSA usually starts before patients fall asleep and the occurrence of UAO, it is not clear whether the efficacy of CPAP in treating OSA is reproducible in unconscious subjects. Therefore, the aims of our study were to determine (1) whether nCPAP is more effective to maintain upper airway patency than FmCPAP during induction of GA and (2) whether the application of CPAP prior to or after induction of GA affects efficacy of mask ventilation with both nasal mask and full face mask. We consider this study highly clinically relevant since currently many providers of emergency mask ventilation and difficult airway management are unable to achieve effective ventilation and alternative approaches are needed.

## Materials and methods

The study protocol was approved by the Massachusetts General Hospital Human Research Committee (Boston, MA, USA), and written informed consent was obtained from all participating subjects.

### Subject enrollment and randomization

**Figure 1:**
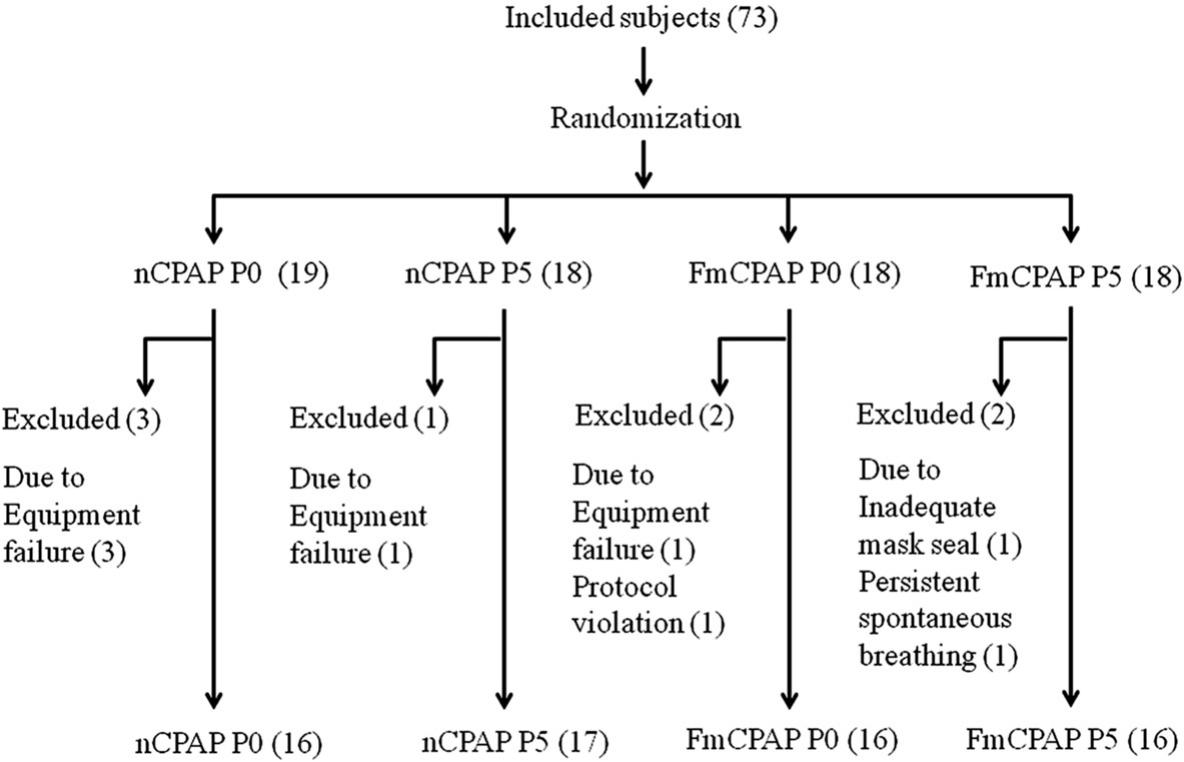
**Subject enrollment and randomization.** Four subjects in group nCPAP and one in group FmCPAP were excluded because of equipment failure. Three subjects in group FmCPAP were excluded because of protocol violations (body mass index >35 kg/m^2^) (n = 1), inadequate seal of face mask (n = 1), and persistent spontaneous breathing (n = 1). Numbers of patients are indicated in parentheses. CPAP, continuous positive airway pressure; FmCPAP, full face mask continuous positive airway pressure/ventilation group; nCPAP, nasal continuous positive airway pressure/ventilation group; P0, initial continuous positive airway pressure level 0 cm H_2_O, P5, initial continuous positive airway pressure level 5 cm H_2_O.

In total, 73 subjects, 18 to 65 years of age, were enrolled in this study from the inpatient main operating rooms of the Massachusetts General Hospital after giving their consent. All subjects who met inclusion and did not have any exclusion criteria, who required GA for elective surgery, who had a preoperative physical status of I or II as defined by the American Society of Anesthesiologists, who were able to breathe through both their nose and mouth, and who had no known contraindications to mask ventilation were approached for consent. Exclusion criteria included major cardiovascular disease, respiratory disease, cerebral vascular disease, gastric-esophageal reflux, or a full stomach, known obstructive sleep apnea, body mass index of greater than 35 kg/m^2^, and the need for emergency surgery. All subjects were randomly assigned into one of four groups by pre-sealed envelope: nCPAP P0, nCPAP P5, FmCPAP P0, and FmCPAP P5, where P0 and P5 represent CPAP 0 and 5 cm H_2_O applied prior to induction of GA. The process of subject selection and randomization is shown in Figure 1.

### Study protocol

**Figure 2:**
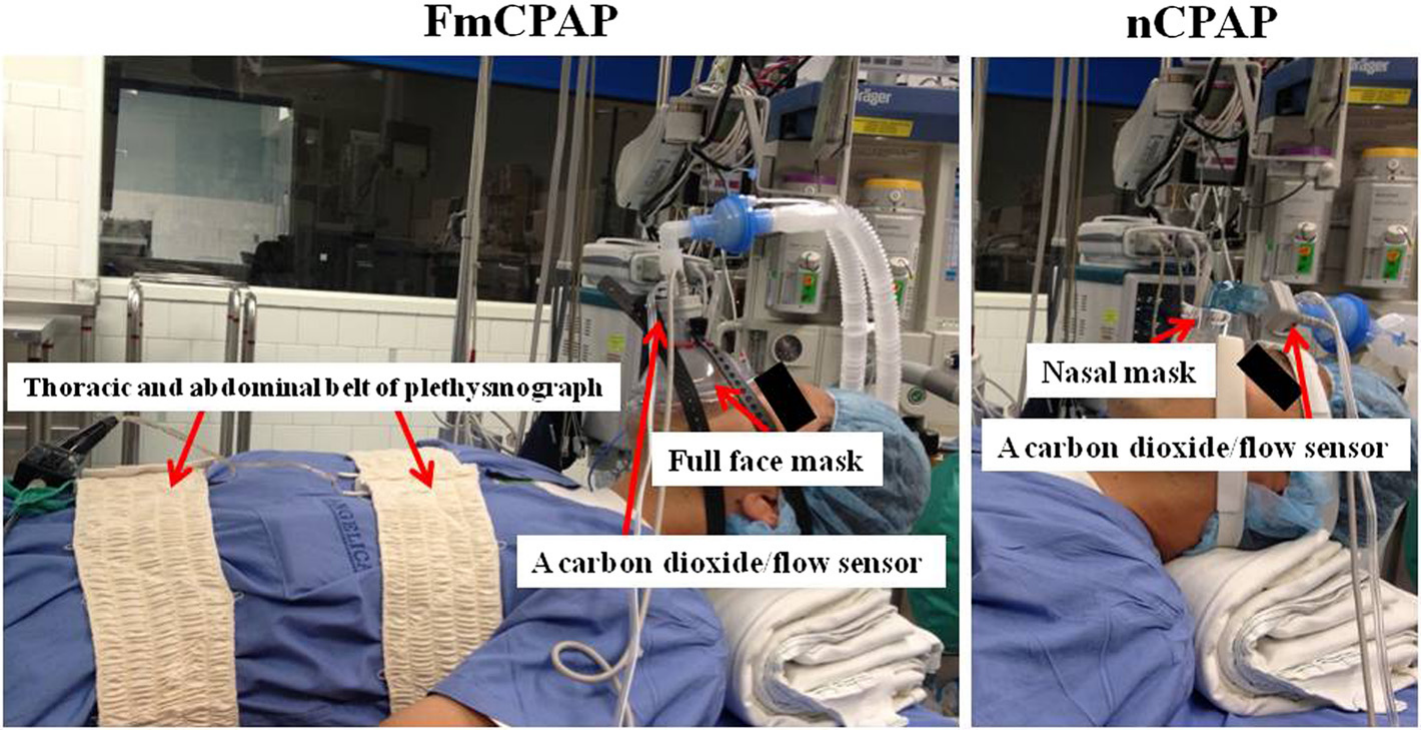
**Experiment set-up.** Left: continuous positive airway pressure via a full face mask and ventilation (FmCPAP). A carbon dioxide (CO_2_)/flow sensor (Novametrix Medical Systems Inc., Wallingford, CT, USA) was placed between the mask and the breathing circuit. The sensor was connected to a non-invasive cardiac output (NICO) monitor (model 7300; Respironics, Murrysville, PA, USA). This sensor allows continual monitoring and recording of the following data: flow waveform, airway pressure, respiratory rate, tidal volume, and exhaled CO_2_ waveform. With the respiratory inductance plethysmograph (Respitrace Calibrator; Ambulatory Monitoring, Inc., Ardsley, NY, USA), the rib cage band was placed at the level of the nipples and the abdominal band at the level of the upper abdomen. Right: continuous positive airway pressure via a nasal mask and ventilation (nCPAP). The equipment is the same as with FmCPAP, except for the nasal mask.

Once patients were placed on the operating room table, electrocardiogram, non-invasive blood pressure, and transcutaneous oxyhemoglobin saturation monitoring were applied. A properly sized mask, full face mask, or nasal mask, based on randomization (nasal mask: Contour Deluxe^®^; Respironics, Murrysville, PA, USA, and full face mask: Ultra-Seal^®^; Baxter, Deerfield, IL, USA), was placed and secured by head straps. We used a head strap in both the nCPAP and FmCPAP groups to avoid the bias introduced by the operator’s technique of mask holding. Mask seal was checked by investigators ensuring that there was no significant air leak at 25 cm H_2_O applied peak inspiratory pressure with the anesthesia machine (Dräger Narkomed 6000 Anesthesia Machine System^®^; Dräger, Lübeck, Germany). To measure and record the airway flow, airway pressure, tidal volume, and exhaled CO_2_, we used a carbon dioxide/flow sensor (Novametrix Medical Systems Inc., Wallingford, CT, USA) which was connected to a non-invasive cardiac output (NICO) monitor (model 7300; Respironics Corp., Murrysville, PA) and respiratory inductance plethysmograph (Respitrace Calibrator; Ambulatory Monitoring, Inc., Ardsley, NY, USA), of which the rib cage band was placed at the level of the nipples and the abdominal band at the level of the upper abdomen. All pieces of equipment used in this study are shown in Figure 2. Because we aimed to simulate a scenario in which airway management was not optimized when emergency ventilation was needed or difficult mask ventilation occurred, the subject’s head was placed on a pillow and elevated about 10 cm from the operating room table, but no backward head tilt, jaw thrust, or mouth closing was performed. Airway patency was indirectly assessed by measuring expired tidal volume (Vte) by using the plethysmograph at a given peak inspiratory pressure over PEEP (PIP/PEEP) [16].

In group nCPAP P0, patients were allowed to breathe spontaneously through the nasal mask connected to an anesthesia machine before induction of GA. They were pre-oxygenated with 100% O_2_ until their fraction of expired oxygen (FeO_2_) was greater than 80% before the beginning of the study. Then, GA was induced as usual with intravenous propofol (2 mg/kg), fentanyl (2-5 μg/kg), and midazolam (0.02-0.05 mg/kg). Maintenance of GA depth was achieved by intravenous bolus of propofol, and the Bispectral Index (BIS) reading was kept from 40 to 60, which was the targeted level of sedation during GA. When the patient was apneic, as evidenced by no flow and no breathing effort, ventilation was initiated with pressure control ventilation at a rate of 10 breaths/min, fraction of inspired oxygen 1.0, and PIP/PEEP 20/0 cm H_2_O, then 20/5 cm H_2_O, and finally 20/10 cm H_2_O each applied for 1 min.

**Figure 3:**
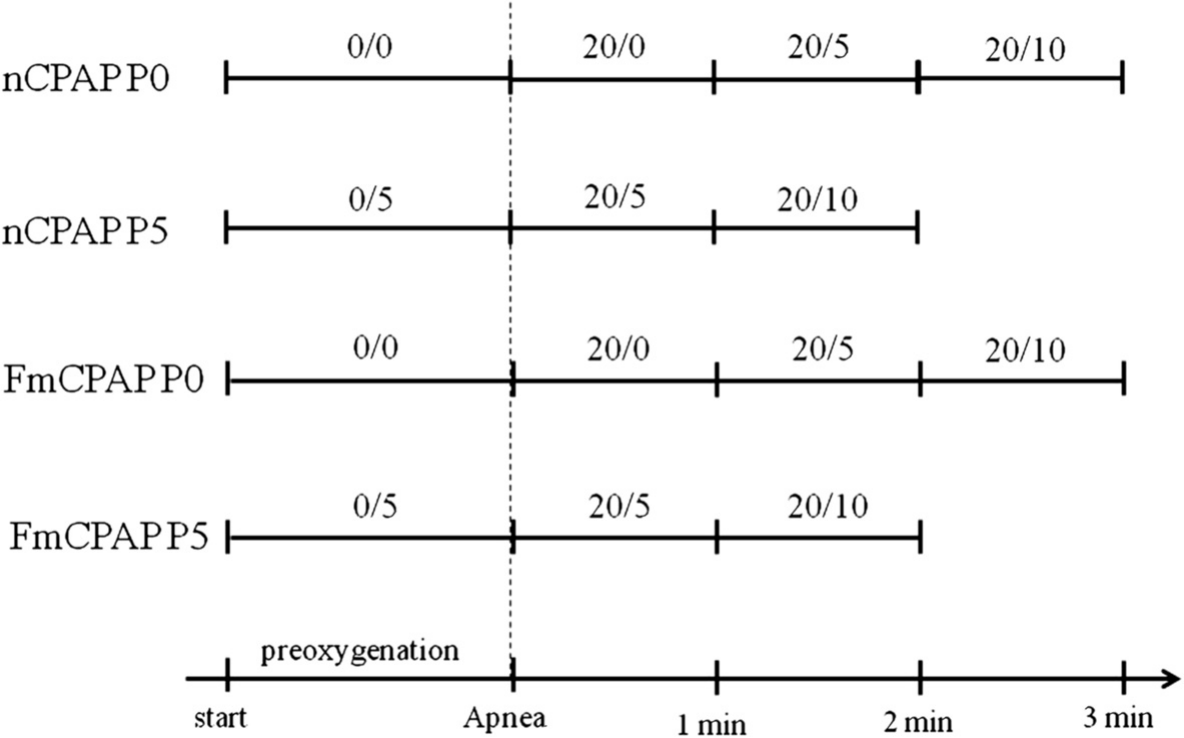
**Airway pressure protocol after randomization.** 20/0, 20/5, and 20/10 represent peak inspiratory pressure (cm H_2_O)/positive end-expiratory pressure (cm H_2_O). FmCPAP, continuous positive airway pressure via a full face mask and ventilation, where P0 and P5 represent continuous positive airway pressure 0 and 5 cm H_2_O applied prior to induction of general anesthesia; nCPAP, continuous positive airway pressure via a nasal mask and ventilation.

In group nCPAP P5, patients received nCPAP at a PEEP of 5 cm H_2_O before induction of GA. GA was induced and maintained as described in group nCPAP P0. After becoming apneic, patients were ventilated with pressure control ventilation at a rate of 10 breaths/min, fraction of inspired oxygen 1.0, and PIP/PEEP 20/5 cm H_2_O and then 20/10 cm H_2_O. In group FmCPAP P0 and FmCPAP P5, patients received the same ventilation settings as in group nCPAP P0 and nCPAP P5, respectively, through a full face mask instead of nasal mask. The airway pressure protocol for each group is shown in Figure 3. During the ventilation phase after anesthetic induction, ventilation was maintained through the randomized mask. In each group, if no adequate ventilation could be generated, as evidenced by observation of end-tidal carbon dioxide (ETCO_2_) at the starting pressure level or of the amplitude changes of the plethysmograph at least after four breaths, the next level of pressure protocol was applied until a level of 20/10 cm H_2_O was achieved. If adequate ventilation was obtained, we continued ventilation for 1 min. If at 20/10 cm H_2_O, no ventilation could be generated, mouth closure in the nCPAP group and head tilt in the FmCPAP group were applied and ventilated for one or two breaths to avoid hypoxemia. And then the routine airway management was initiated. At any time, if the blood oxygen saturation (SpO_2_) decreased to 92% or the ETCO_2_ increased to more than 50 mm Hg, the study was terminated and routine airway management was resumed.

Upon completion of the study protocol, muscle relaxant was given as normally done. Endotracheal tube or laryngeal mask airway placement was pursued as usual. Once the subject was intubated, the calibration curve for the plethysmograph was generated as previously described [17]. Specifically, the calibration curve was created by using the measured Vte by NICO and the amplitude changes of the plethysmograph readings during mechanical inspiration and expiration provided by the anesthesia ventilator. Then the Vte during each pressure setting was calculated retrospectively by using the calibration curve from each subject.

At each pressure setting, 10 breaths were collected and the last 5 breaths were analyzed for determination of Vte as the first few breaths were not at steady state. Effective tidal volume was determined by the number of breaths with Vte being greater than the estimated anatomic dead space (2.2 mL/kg ideal body weight) [18]. All ventilation parameters, including respiratory rate, flow waveform, and peak inspiratory airway pressure, were recorded with the NICO monitor. The delta pressure (delta P) during inspiration, defined as the pressure gradient measured from the baseline airway pressure to the maximum airway pressure at the end of the breath, was calculated by determining the pressure applied during inspiration.

The primary endpoints of this study were the Vte and the rate of effective tidal volume (Vte > estimated anatomical dead space). Owing to the air leak, the airway pressure varied in each subject even though the target airway pressure was the same. Therefore, we added the values of Vte/delta P to determine differences in airway patency.

### Statistical analysis

Based on the data obtained from our previous study [14] using nasal mask versus mouth piece and nasal mask interface, the volume of CO_2_ removed per breath divided by the PIP (ETCO_2_/PIP) was used to calculate the sample size of this study. A sample size of 64 allowed us to detect a difference of 0.7 times the standard deviation or larger between the two ventilatory methods with 80% power. We planned to enroll 80 subjects, assuming a failure rate of 20%. To minimize the potential bias, the data analysis was performed blind and the person who calculated the Vte and airway pressure was unaware which treatment, nCPAP versus FmCPAP, the subject received. Variables were first tested for normal distribution with the Shapiro-Wilk test. Data are presented as the mean ± standard deviation or, if not normally distributed, as median value (interquartile range). Analysis of variance was performed by using the Friedman test or Kruskal Wallis *H* test. Wilcoxon *t* test with Bonferroni correction or Mann-Whitney *U* test with Bonferroni correction was performed as a *post hoc* test for multiple comparisons. Two-by-two comparisons were obtained by using the Wilcoxon signed rank test. Mann-Whitney *U* test was used to evaluate non-paired measurements. Categorical variables were analyzed by using the chi-square test. Statistical analysis was performed with a commercially available statistical package (SPSS, version 12.0; SPSS Inc., Chicago, IL, USA), and a *P* value of less than 0.05 was considered significant.

## Results

In total, 73 subjects were randomly assigned (Figure 1). There were no cases of difficult mask ventilation [19]. Four of fifty-three subjects were intubated by portable video laryngoscope for educational purposes. Two subjects in the nCPAP group (one in the nCPAP P0 and one in the nCPAP P5 group) and 25 subjects in FmCPAP group (14 in FmCPAP P0 and 11 in FmCPAP P5 group) showed zero Vte after four breaths. No subjects showed a SpO_2_ of less than 92% or the ETCO_2_ of greater than 50 mm Hg. Demographic data, including Cormack and Lehane classification grade [20], are summarized in Table 1. There were no differences in the demographic data between groups.

**Table 1:** Demographic data

	**NM group (n = 37)**	**FM group (n = 36)**	***P *** **value**
Age, y	47 ± 11	49 ± 12	0.56
Gender, male/female	14/23	14/22	0.93
Height, cm	169 ± 12	166 ± 9	0.28
Weight, kg	76.2 ± 14.4	72.5 ± 14.9	0.29
BMI, kg/m^2^	26.7 ± 3.9	26.8 ± 4.8	0.92
Airway management			
ETT/LMA, n	25/12	28/8	0.47
Cormack and Lehane grade			
Grade 1, n	22	25	0.77
Grade 2, n	1	1	1.00
Video laryngoscope, n	2	2	1.00

Values are mean ± standard deviation. BMI, body mass index; ETT, endotracheal tube; FM, full face mask; LMA, laryngeal mask airway; NM, nasal mask.

**Figure 4:**
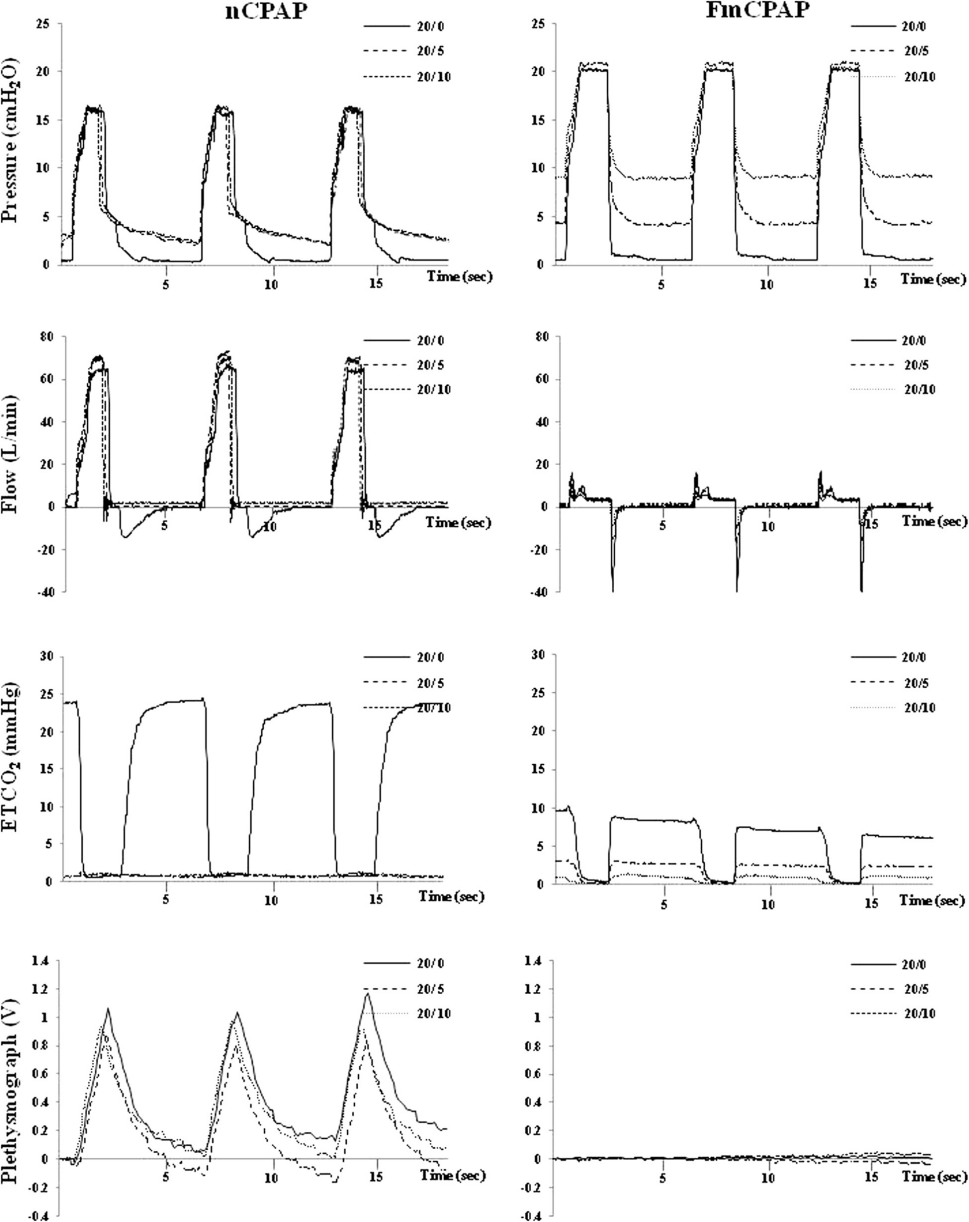
**Representative waveforms of airway pressure, airway flow, end tidal carbon dioxide, and signal of plethysmograph.** Airway pressure: In the nCPAP group, PIP did not reach the target pressure during any of the ventilator settings (PIP/PEEP of 20/0, 20/5, and 20/10 cm H_2_O) and PEEP was not maintained at the set level during 20/5 and 20/10 cm H_2_O. In the FmCPAP group, airway pressure reached and maintained the target pressure. Airway flow: In the nCPAP group, while airway flow during inspiration was almost the same among the three ventilator settings, expired flow was detected in only the 20/0 setting. In the FmCPAP group, airway flow was not detected or very low values were detected during both inspiration and expiration at all three ventilator settings. ETCO_2_: In the nCPAP group, ETCO_2_ was detected in only the 20/0 settings. In the FmCPAP group, ETCO_2_ was not detected or very low values were detected with each of the three ventilator settings. Plethysmograph: In the nCPAP group, the signal of plethysmograph was detected with each of three ventilator settings. In the FmCPAP group, the plethysmograph was not detected with any of the three ventilator settings. The values of plethysmograph are presented as the mean values of the movement of rib cage and abdomen. The mean value was calculated by using the sum of readings from the movement of the rib cage and abdomen and then divided by 2. ETCO_2_, end tidal carbon dioxide; FmCPAP, continuous positive airway pressure via a full face mask and ventilation; nCPAP, continuous positive airway pressure via a nasal mask and ventilation.

Figure 4 shows representative waveforms of airway pressure, respiratory flow, end tidal CO_2_ (ETCO_2_), and plethysmograph in both the nCPAP and FmCPAP groups. In the nCPAP group, airway pressure, both peak inspiratory pressure and PEEP, could not be maintained at the target pressure, because of the air leak from the mouth. Essentially, the data obtained at PEEP 5 and 10 cm H_2_O were almost the same. Although airway flow and ETCO_2_ values were not detected or very low values identified because of air leak, the amplitude of the plethysmograph waveforms indicated successful ventilation. In the FmCPAP group, airway pressure, both peak inspiratory pressure and PEEP, was almost maintained at the target pressure. However, airway flow and ETCO_2_ were not detected or very low values identified and the amplitude of plethysmograph waveform indicated no ventilation.

### Vte with nCPAP and FmCPAP application

There were significant differences in Vte among the four groups (*P* <0.01). The median Vte values in nCPAP P0 (5.7 mL/kg) and P5 (7.7 mL/kg) were larger than those of FmCPAP P0 (0 mL/kg) and P5 (0 mL/kg) (*P* <0.01). But there was no difference in Vte between nCPAP P0 and P5 (5.7 vs. 7.7 mL/kg, *P* = 0.12) or between FmCPAP P0 and P5 (0 vs. 0 mL/kg, *P* = 0.25).

**Figure 5:**
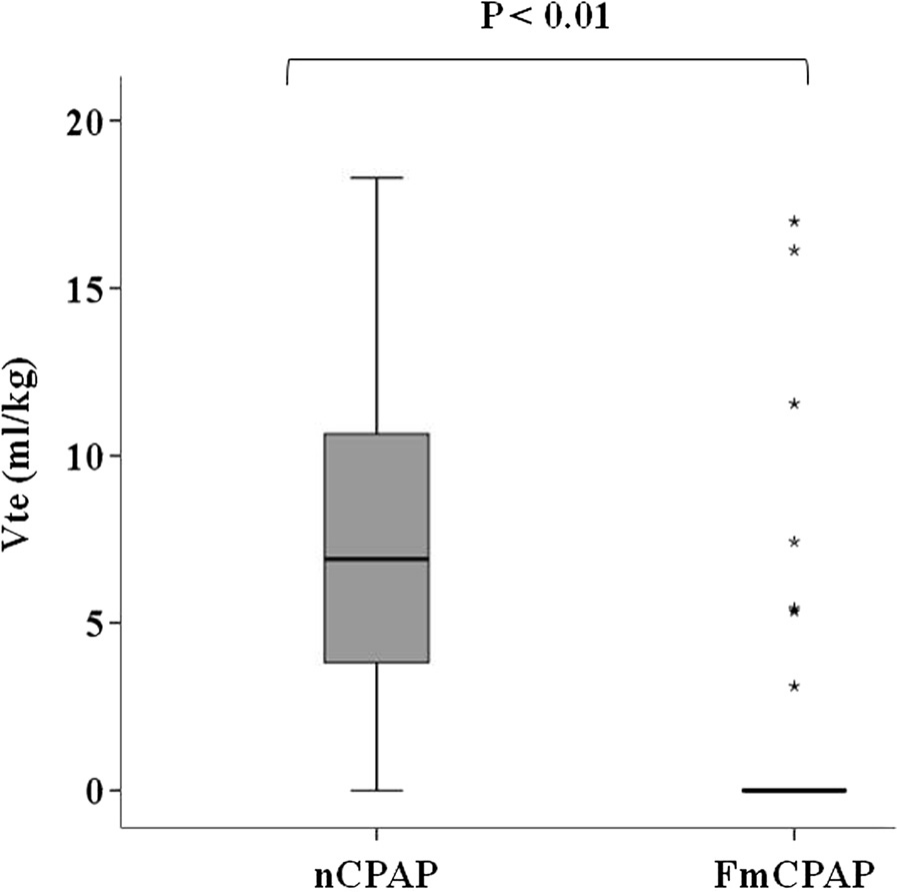
**Effects of nCPAP and FmCPAP on expired tidal volume.** Each box represents the interquartile range between the 25th and the 75th percentiles, with the median value shown as a horizontal line within the box. Vertical bars represent the maximum and minimum values except for outliers. Outliers identified with a star are any data which lie more than 3.0 times the box length beyond the lower or higher edges of the box. FmCPAP, continuous positive airway pressure via a full face mask and ventilation; nCPAP, continuous positive airway pressure via a nasal mask and ventilation; Vte, expired tidal volume (in milliliters per kilogram of ideal body weight).

The median Vte in the nCPAP group was larger than that of the FmCPAP group (6.9 vs. 0 mL/kg; *P* <0.01) (Figure 5). The rate of effective tidal volume (Vte ≥ anatomical dead space) was higher in the nCPAP group compared with the FmCPAP group (29/33: 87.9% vs. 7/32: 21.9%; *P* <0.01). In the nCPAP group, all subjects who failed to achieve effective tidal volume were able to achieve larger Vte simply by having their mouth closed by the investigator (median Vte: 0 vs. 5.8 mL/kg). In the FmCPAP group, all subjects who failed to achieve effective tidal volume were able to obtain larger Vte with backward head tilt and jaw thrust (median Vte: 0 vs. 8.3 mL/kg).

### Application of CPAP prior to or after induction of GA

Application of CPAP prior to induction did not significantly affect the rates of effective tidal volume (nCPAP pre- vs. post- GA induction; 16/17: 94.1% vs. 13/16: 81.3%, *P* = 0.36) (FmCPAP pre- vs. post- GA induction; 5/16: 31.3% vs. 2/16: 12.5%, *P* = 0.42) and the median Vte in both approaches (nCPAP: pre- vs. post- GA induction; 7.9 vs. 5.8 mL/kg, *P* = 0.07) (FmCPAP: pre- vs. post- GA induction; 0 vs. 0 mL/kg, *P* = 0.11). Application of CPAP after induction of GA generated a higher rate of effective tidal volume (13/16: 81.3% vs. 2/16: 12.5%, *P* <0.01) and larger median Vte (5.8 vs. 0 mL/kg, *P* <0.01) in the nCPAP group than the FmCPAP group.

### Delta P and Vte/delta P in each ventilator setting

**Figure 6:**
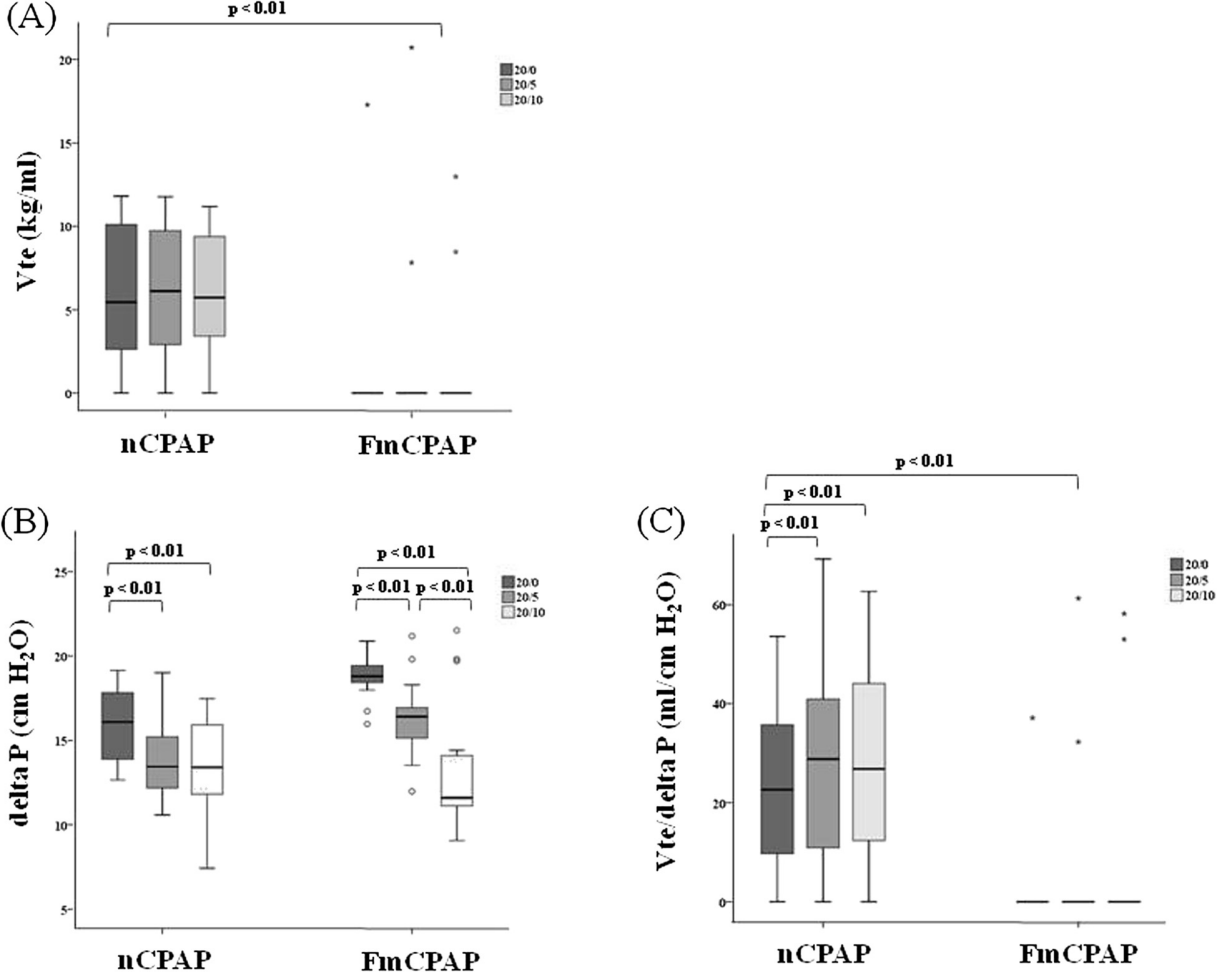
**Effects of each ventilator setting on Vte, delta pressure, and Vte/delta pressure in both nCPAP and FmCPAP. (A)** Expired tidal volume obtained with nCPAP and FmCPAP. **(B)** Delta pressure measured in nCPAP and FmCPAP. **(C)** Vte/delta P in nCPAP and FmCPAP. Each box represents the interquartile range between the 25th and the 75th percentiles, with the median value shown as a horizontal line within the box. Vertical bars represent the maximum and minimum values except for outliers. Outliers identified with a circle are any data values which lie greater than 1.5 times the box length beyond the lower or higher edges of the box and within 3.0 times the box length. Outliers identified with a star are any data which lie more than 3.0 times the box length beyond the lower or higher edges of the box; 20/0, 20/5, and 20/10 represent peak inspiratory pressure (PIP) (cm H_2_O)/positive end-expiratory pressure (PEEP) (cm H_2_O). delta P, delta pressure; FmCPAP, continuous positive airway pressure via a full face mask and ventilation; nCPAP, continuous positive airway pressure via a nasal mask and ventilation; Vte, expired tidal volume (in milliliters per kilogram of ideal body weight).

Figure 6 shows Vte, Delta P, and Vte/delta P in each ventilator setting in both nCPAP and FmCPAP. There were no significant differences in Vte at each ventilator setting in the nCPAP and the FmCPAP group (Panel 6a). In the FmCPAP group, as PEEP levels increased, delta P decreased (Panel 6b). In the nCPAP group, delta P at PEEP 0 cm H_2_O was larger than that of PEEP 5 and 10 cm H_2_O while there was no difference in delta P between PEEP 5 and 10 cm H_2_O (Panel 6b). The median baseline pressure of nCPAP was less than that of FmCPAP (nCPAP: 0.2, 3.0, and 2.8 cm H_2_O, vs. FmCPAP: 0.5, 4.3, and 9.2 cm H_2_O at PIP/PEEP of 20/0, 20/5, and 20/10 cm H_2_O, respectively; *P* <0.01). In the nCPAP group, although there were no differences in Vte in each PEEP settings, Vte/delta P values at PEEP 5 and 10 cm H_2_O were higher than that of PEEP 0 cm H_2_O (Panel 6c).

## Discussion

Our study conducted on unconscious subjects demonstrated that (1) nCPAP increased the rate of effective tidal volume and Vte compared with FmCPAP, (2) the application of CPAP prior to induction of GA did not affect Vte in either approach, and (3) in the nCPAP group, although there were no differences in Vte at each PEEP settings, Vte/delta P at PEEP 5 and 10 cm H_2_O were higher than that of PEEP 0 cm H_2_O. These results suggested that application of nCPAP maintains upper airway patency and may produce more effective ventilation than FmCPAP.

Once an individual loses consciousness, particularly in the supine position, the upper airway is inevitably obstructed [8,21]. The mechanisms of UAO in patients with OSA or during GA are multifactorial. The main issues are a reduction in tone of the dilator muscles of the pharynx, gravity pulling the tongue and soft palate downward, and a reduction in lung volume [22,23]. In this study, nCPAP increased the rate of effective tidal volume and Vte compared with FmCPAP. One possible reason was that nCPAP creates a positive pressure during both inspiration and expiration in the nasopharyngeal cavities which functions as a pneumatic splint that prevents UAO by pushing the soft palate and the tongue forward and away from the posterior pharyngeal wall [10,22]. Because the soft palate lies behind the tongue, the pressure gradient between the nasopharyngeal and oropharyngeal cavities pushes the soft palate and tongue forward, opening the airway [14]. nCPAP also increases lung volume and dilates the pharyngeal cavity [23]. FmCPAP applies positive pressure in both the nasopharyngeal and oropharyngeal cavities without generating a pressure gradient, allowing gravity to displace the tongue and soft palate backward, causing airway obstruction [14].

Even when the delta P was not kept constant, there were no significant differences of Vte at any given ventilator setting (PIP/PEEP of 20/0, 20/5, and 20/10 cm H_2_O) and Vte/delta P at PEEP 5 and 10 cm H_2_O were higher than at PEEP 0 cm H_2_O in the nCPAP group. In other words, even when the delta P is reduced, Vte increased as PEEP increased. Previous studies using magnetic resonance imaging demonstrated that CPAP reduced anesthesia-induced upper airway narrowing in both adults and infants [24,25]. In this study, we did not intend to determine the optimal level of PEEP required for maintaining airway patency, so we arbitrarily measured the Vte at three levels of PEEP, 0, 5, and 10 cm H_2_O. Owing to the air leak from the mouth, baseline airway pressure was not maintained as the target pressure, but nCPAP most likely reduced upper airway narrowing in this study. Because the air leak with FmCPAP was much less than that with nCPAP, baseline pressure during FmCPAP was significantly greater than that of nCPAP at any given level of PEEP. However, nCPAP produced larger Vte per unit of delta P than FmCPAP.

During FmCPAP, in about 80% of subjects, the Vte was so small that our equipment was unable to detect the tidal volume. We did not perform backward head tilt or jaw thrust as anesthesia care and emergency care providers usually do. If jaw lift and head tilt were performed, the difference in the Vte between nCPAP and FmCPAP might be smaller than what we observed in this study. Further clinical studies will be needed to confirm whether nCPAP will be superior to FmCPAP with optimal airway management. In this study, we did not apply any head position change or mouth closure to optimize upper airway management, to avoid the bias introduced by the operator’s technique of mask ventilation. Furthermore, we intended to test whether nasal mask ventilation was superior to full face mask ventilation when airway management is not optimized as frequently occurs when emergency ventilation is needed or difficult mask ventilation occurs. It has been reported that more than 50% of emergency medical technicians and 84% of emergency nurses before training were not able to ventilate mannequins efficiently in simulated emergency situations [5,6]. It is our impression that many caregivers do not place the head in an optimal position when providing full face mask ventilation, especially in emergency situations. Therefore, we conducted this study to simulate non-optimized mask ventilation; no jaw thrust or backward head tilt was performed. Because we did not intentionally close the mouth when nCPAP was applied, the majority of subjects had significant leaks through the mouth, but they still achieved effective tidal volume. Because all subjects who failed to achieve effective tidal volume were able to achieve larger Vte simply by closing their mouths, we believe that the success rate of nCPAP may be much higher than what we observed in this study if the mouth was closed. In the FmCPAP group, the range of Vte was large and six outliers were identified out of 36 patients. We were unable to find differences among subjects with successful and unsuccessful ventilation. Body mass index, condition of mask seal, pressure profile of airway, and Cormack and Lehane grades were similar among subjects with successful and unsuccessful ventilation. It is hard to predict the difficult airway even though we used the algorithms of pre-operative assessment of the difficult airway [26]. It is possible that the airways of these outliers were simply more patent and a larger Vte could be delivered. As we did not measure the caliber of the oropharyngeal cavity, we cannot speculate what caused the increased airway patency with FmCPAP in these subjects.

It was well established that once UAO in patients with OSA occurs, it requires much higher inspiratory pressure to open the collapsed airway and the end-expiratory pressure requirement to maintain the airway patent is lower after the obstruction is overcome [27-29]. We observed that the application of CPAP prior to induction of GA did not affect Vte in either approach. There has been no study to investigate the effects of CPAP levels applied before induction of GA on airway patency. We believe that the application of CPAP prior to induction of GA improved ventilation by maintaining the airway patent, but the amplitude of the improvement was not as dramatic as that of OSA. This is a critical issue since in clinical practice in the majority of situations requiring emergency ventilation application would occur after UAO developed.

There are several limitations to this study. First, we used head straps with both the nasal mask and face mask to avoid the bias introduced by the operator’s technique of mask holding. However, there was the possibility of downward displacement of the chin by the head strap in patients wearing the face masks, and as a result the mandibular position of the FmCPAP group was different from that of the nCPAP group. We continually observed the position of the head and mandible to ensure that the head stayed in the same position. During this process, we did not observe the mandible being pushed down after induction of anesthesia. In addition, we cannot find any documentation that this is a regular concern during full face mask ventilation. In our previous study [14], we used head straps for both nasal and oral mask application. Although the head strap for oral mask remained in place during nasal mask ventilation, nasal mask ventilation was still more effective than combined nasal and oral mask ventilation. This indicated that the head strap for face mask did not markedly affect airway collapsibility. Second, we determined effectiveness of ventilation with a head strap holding the nasal and full face mask rather than testing hand-holding the masks. The nasal mask used in the study was much smaller than the full face mask. Therefore, holding the nasal mask should be much easier than the full face mask. In addition, because application of the nasal mask is on a rigid plane of the upper jaw, it is much easier to obtain an adequate mask seal. Third, we did not control for the pharyngeal dilator muscle activities during this study. Instead, we intended to keep the depth of sedation constant by using integrated electroencephalography. Since we conducted a randomized controlled study, we assumed that effects of pharyngeal dilator muscle activities were equally affected in both groups and this would not affect outcome. Fourth, we observed larger air leaks with nCPAP than with FmCPAP. In clinical practice, the air leak from the mouth during nCPAP may be challenging because maximal fresh gas flow is limited with anesthesia machines and during field emergency mask ventilation. However, leaking from the mouth can be eliminated with mouth closure. Fifth, we evaluated the effectiveness of ventilation in a suboptimized airway condition. We did not compare the two approaches during an optimized airway and as a result we do not know whether nCPAP is superior to FmCPAP when the airway is optimized. Sixth, the ETCO_2_/PIP was used to calculate sample size based upon data from our previous study [14]. However, because of large air leaks during nCPAP, ETCO_2_ in most of the subjects in this group was zero or close to zero. These subjects were adequately ventilated as evidenced by plethysmograph. As a result, we were unable to use ETCO_2_/PIP as our primary outcome variable. Finally, because this study was performed in healthy subjects with unknown risk of UAO, it is unclear whether our results are applicable to subjects with UAO or in emergency mask ventilation.

## Conclusions

nCPAP was more effective in maintaining upper airway patency in unconscious subjects and produced more effective tidal volume than FmCPAP. Application of nCPAP even after UAO developed was more effective than FmCPAP and generated nearly 100% effective tidal volume simply by closing the mouth. Further clinical studies will be needed to confirm whether nCPAP will be superior to FmCPAP with optimal airway management.

## Key messages

• Continuous positive airway pressure (CPAP) and ventilation via the nasal mask (nCPAP) is more effective in maintaining upper airway patency than full face mask CPAP and ventilation (FmCPAP).

• Application of CPAP prior to upper airway obstruction does not affect Vte in either nCPAP or FmCPAP.

## Abbreviations

CPAP: continuous positive airway pressure; ETCO2: end-tidal carbon dioxide; FmCPAP: continuous positive airway pressure and ventilation via the full face mask; GA: general anesthesia; nCPAP: continuous positive airway pressure and ventilation via the nasal mask; NICO: non-invasive cardiac output; OSA: obstructive sleep apnea; PEEP: positive end-expiratory pressure; PIP: peak inspiratory pressure; SpO2: blood oxygen saturation; UAO: upper airway obstruction; Vte: expired tidal volume.

## Competing interests

RMK is a consultant for Covidien (Dublin, Ireland), has received research grants from Covidien, and has received honorarium for lecturing from Maquet (Rastatt, Germany). The other authors declare that they have no competing interests.

## Authors’ contributions

JO, RMK, and YJ designed and conducted the study, collected and analyzed the data, and wrote the manuscript. QL conducted the study and analyzed the data. WRK, JW, ASS, and PGH helped enroll the patients, followed protocol, and collected the data. All authors read and approved the final manuscript.

## Authors’ information

JO and QL are first authors.
